# How do lizard niches conserve, diverge or converge? Further exploration of saurian evolutionary ecology

**DOI:** 10.1186/s12862-021-01877-8

**Published:** 2021-07-30

**Authors:** Nicolás Pelegrin, Kirk O. Winemiller, Laurie J. Vitt, Daniel B. Fitzgerald, Eric R. Pianka

**Affiliations:** 1 Facultad de Ciencias Exactas, Físicas y Naturales, Centro de Zoología Aplicada, and Consejo Nacional de Investigaciones Científicas y Técnicas (CONICET), Instituto de Diversidad y Ecología Animal (IDEA), Universidad Nacional de Córdoba, Rondeau 798, X5000AVP Córdoba, Argentina; 2 Department of Ecology and Conservation Biology, Texas A&M University, College Station, TX 77843-2258 USA; 3 Sam Noble Museum and Department of Biology, University of Oklahoma, Norman, 73072 USA; 4 U.S. Geological Survey, Eastern Ecological Science Center, Kearneysville, WV 25430 USA; 5 Department of Integrative Biology, University of Texas, Austin, TX 78712-0253 USA

**Keywords:** Adaptive divergence, Evolutionary convergence, Functional group, Periodic table of niches, Phylogenetic niche conservatism, Sauria

## Abstract

**Background:**

Environmental conditions on Earth are repeated in non-random patterns that often coincide with species from different regions and time periods having consistent combinations of morphological, physiological and behavioral traits. Observation of repeated trait combinations among species confronting similar environmental conditions suggest that adaptive trait combinations are constrained by functional tradeoffs within or across niche dimensions. In an earlier study, we assembled a high-resolution database of functional traits for 134 lizard species to explore ecological diversification in relation to five fundamental niche dimensions. Here we expand and further examine multivariate relationships in that dataset to assess the relative influence of niche dimensions on the distribution of species in 6-dimensional niche space and how these may deviate from distributions generated from null models. We then analyzed a dataset with lower functional-trait resolution for 1023 lizard species that was compiled from our dataset and a published database, representing most of the extant families and environmental conditions occupied by lizards globally. Ordinations from multivariate analysis were compared with null models to assess how ecological and historical factors have resulted in the conservation, divergence or convergence of lizard niches.

**Results:**

Lizard species clustered within a functional niche volume influenced mostly by functional traits associated with diet, activity, and habitat/substrate. Consistent patterns of trait combinations within and among niche dimensions yielded 24 functional groups that occupied a total niche space significantly smaller than plausible spaces projected by null models. Null model tests indicated that several functional groups are strongly constrained by phylogeny, such as nocturnality in the Gekkota and the secondarily acquired sit-and-wait foraging strategy in Iguania. Most of the widely distributed and species-rich families contained multiple functional groups thereby contributing to high incidence of niche convergence.

**Conclusions:**

Comparison of empirical patterns with those generated by null models suggests that ecological filters promote limited sets of trait combinations, especially where similar conditions occur, reflecting both niche convergence and conservatism. Widespread patterns of niche convergence following ancestral niche diversification support the idea that lizard niches are defined by trait-function relationships and interactions with environment that are, to some degree, predictable and independent of phylogeny.

**Supplementary Information:**

The online version contains supplementary material available at 10.1186/s12862-021-01877-8.

## Background

Environmental conditions on Earth are repeated in non-random patterns. Deserts are located about 30° North and South, rainforests occur mainly in tropical regions, and temperatures decrease from the Equator to the poles as well as from low to high altitudes. Similar combinations of environmental conditions are found in distant places and even during different geological periods. Repeated templates of environmental conditions often coincide with consistent sets of morphological, physiological and behavioral traits among species [1–5], patterns that may or may not result from similar processes and evolutionary sequences.

Species from different regions can adapt to similar environmental conditions by evolving similar traits through evolutionary convergence or by retaining ancestral traits [niche conservatism, [2, 3] and remaining within, or dispersing into areas where those traits are adaptive. Both convergence and conservatism have been recognized as equally important in explaining similarity in species traits over relatively large spatial and temporal scales [3], including similarities in behavior, morphology, physiology, and even genes [6–9, among others].

Repeated patterns in form-function relationships suggest the possibility that successful combinations of traits may be constrained by tradeoffs across or within niche dimensions. Past research has often found evidence of such constraints along single niche dimensions. For example, empirical evidence of fundamental constraints on life history variation have been shown for plants and animals [10, 11], and the leaf economic spectrum describes well-established tradeoffs in plant metabolic strategies [12]. Empirical evidence for constraints across multiple niche dimensions is more limited, and often focused on geographically or taxonomically restricted datasets [13, 14]. Few studies have tested whether patterns of functional similarity represent constrained evolutionary trajectories on a global scale [i.e. 15].

Most traits have demonstrable relations with aspects of performance and thus are recognized as “functional traits” [16]. Such traits reflect organismal adaptations to the challenges and opportunities posed by the organism’s environment, and thus determine how the organism affects community and ecosystem processes [17]. In animals, functional traits have been used to define life history and behavioral syndromes [4, 5, 17]. In lizards, sets of traits have been analyzed to elucidate how species partition niches along various dimensions [i.e. habitat, food, and time, 18, 19]. Species with similar functional traits—and thus being affected similarly by environmental conditions and/or affecting the ecosystem in a similar manner—can be aggregated into functional groups.

In this study, we analyze a large, global dataset of lizard traits to investigate the evolution of lizard niches and the extent to which trait values and trait combinations are repeated throughout the lizard phylogeny. Consistent patterns of trait association within and between various niche dimensions provide a basis for inference of functional groups arising from universal constraints. Here, we expand the original proposal of Winemiller et al*.* [20] and Pianka et al*.* [19] for organizing species according to trait similarity with respect to five fundamental niche dimensions. Our aim was to answer the following questions: 1—Is there evidence for consistent constraints to lizard functional diversity? 2—What niche dimensions are most important for identifying functional groups? 3—To what extent do ecological and phylogenetic factors influence the composition of functional groups? To address these questions, we tested the overarching hypothesis that lizard species are grouped around evolutionary optima defined by constrained sets of functional traits and influenced by phylogenetic relationships. We combined available information for lizard functional traits and our own extensive and detailed datasets and performed analyses of trait association to identify functional groups and infer ecological and phylogenetic influences on their compositions. This combined dataset included the entire range of habitats and associated environmental conditions occupied by lizards globally as well as species from 80% of the extant lizard families, thus achieving a world-wide representation of lizard functional ecology.

## Results

**Fig. 1:**
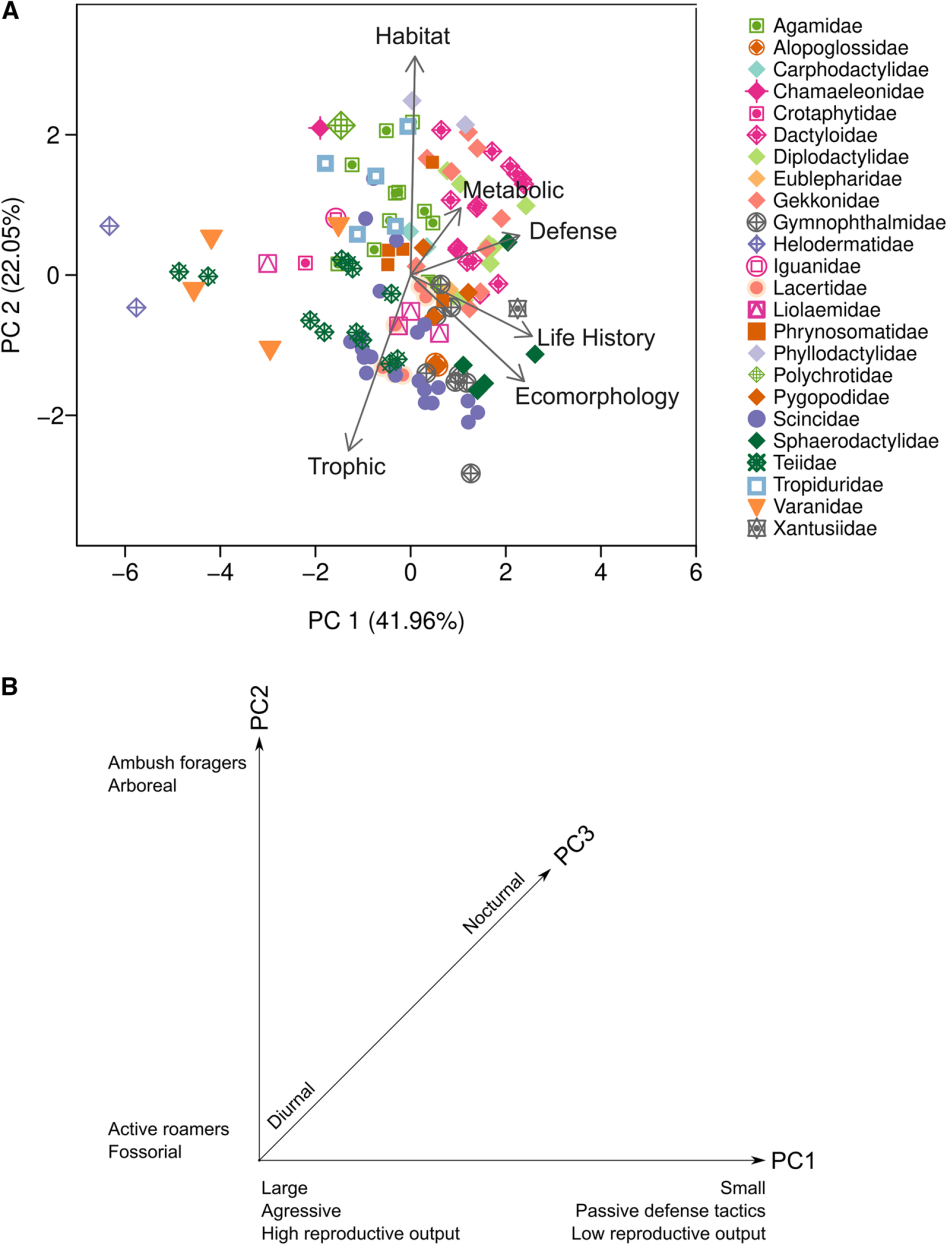
**A** Ordination of 134 lizard species on gradients from principal components analysis (PCA) based on species scores on each of dominant axes generated from six separate principal coordinates analyses (PCoA) performed on functional trait matrices associated with five niche dimensions and ecomorphology. **B** Interpretation of gradients and variables related to each axis

From the six PCoAs performed on Pianka and Vitt’s dataset (134 lizard species and 41 ecological variables, see Methods section) according to five niche dimensions (habitat, trophic, life history, metabolic and defense) and ecomorphology, the first PCoA axis captured the following percentages of the total variation: habitat 50.6%, trophic 42.7%, life history 42.4%, metabolic 84.6%, defense 32.4%, and ecomorphology 74.3%. For the PCA performed using species scores on the first axis from each of these six PCoAs as input data, the first three axes modeled 79.28% of the total variation (Table 1, Fig. 1). Ecomorphology, life history and defense were the dimensions with strongest influence on PCA axis 1. Habitat and trophic dimensions had the strongest influence on PCA axis 2, and the metabolic dimension had the strongest influence on axis 3 (Table 2).

**Table 1:** Percentage of variance captured by the three first axes in the principal coordinates analyses (PCoA) made for each niche dimension, and for the principal component analysis (PCA) made using the functional variables extracted from PCoA

	PC 1	PC 2	PC 3	Total
*PCoA*				
Ecomorphology	74.28	12.39	7.00	93.67
Habitat	50.60	35.11	11.29	97.00
Life history	42.39	29.55	8.44	80.38
Trophic	42.70	16.41	9.62	68.73
Metabolic	84.65	7.69	2.47	94.81
Defense	32.42	23.86	11.23	67.51
PCA	41.96	22.05	15.27	79.28

**Table 2:** Importance of variables to each of the 3 PCA principal components

Variable	PC 1			PC 2			PC 3		
	Contrib (%)	Corr	P	Contrib (%)	Corr	P	Contrib (%)	Corr	P
Ecomorphology	**28.1**	**0.84**	** < 0.0001**	11.32	0.39	< 0.0001	0.09	–	–
Habitat	0.04	–	–	**47.92**	**− 0.8**	** < 0.0001**	10.28	− 0.31	0.0003
Life history	**32.17**	**0.9**	** < 0.0001**	3.75	–	–	3.12	–	–
Trophic	8.37	− 0.46	< 0.0001	**30.94**	**0.64**	** < 0.0001**	0.02	–	–
Metabolic	5.48	0.37	< 0.0001	4.5	–	–	**83.04**	**0.87**	** < 0.0001**
Defense	**25.84**	**0.81**	** < 0.0001**	1.57	–	–	3.45	–	–

The most important functional variables for each component are in bold. Correlation (Corr) and significance (P) of variables with a contribution (Contrib) < 5% were not calculated

The observed 6-dimensional functional hypervolume occupied by lizards was significantly smaller than hypervolumes obtained for each of the four null models, indicating that observed hypervolumes represent a constrained portion of plausible functional trait combinations. The observed hypervolume was closest to that estimated under Null Model 4, which maintained the correlation structure between functional variables and assumed species were normally distributed about the center of functional space (Table 3).

**Table 3:** Comparison between functional hypervolumes obtained from the distribution of species in a 6D functional hyperspace, and those from null models

	Volume	Ratio	P-value
Observed	105.75	–	–
Null Model 1	1129.28	90.56	0.001
Null Model 2	451.34	76.43	0.001
Null Model 3	381.07	72.07	0.001
Null Model 4	159.37	32.76	0.001

**Fig. 2:**
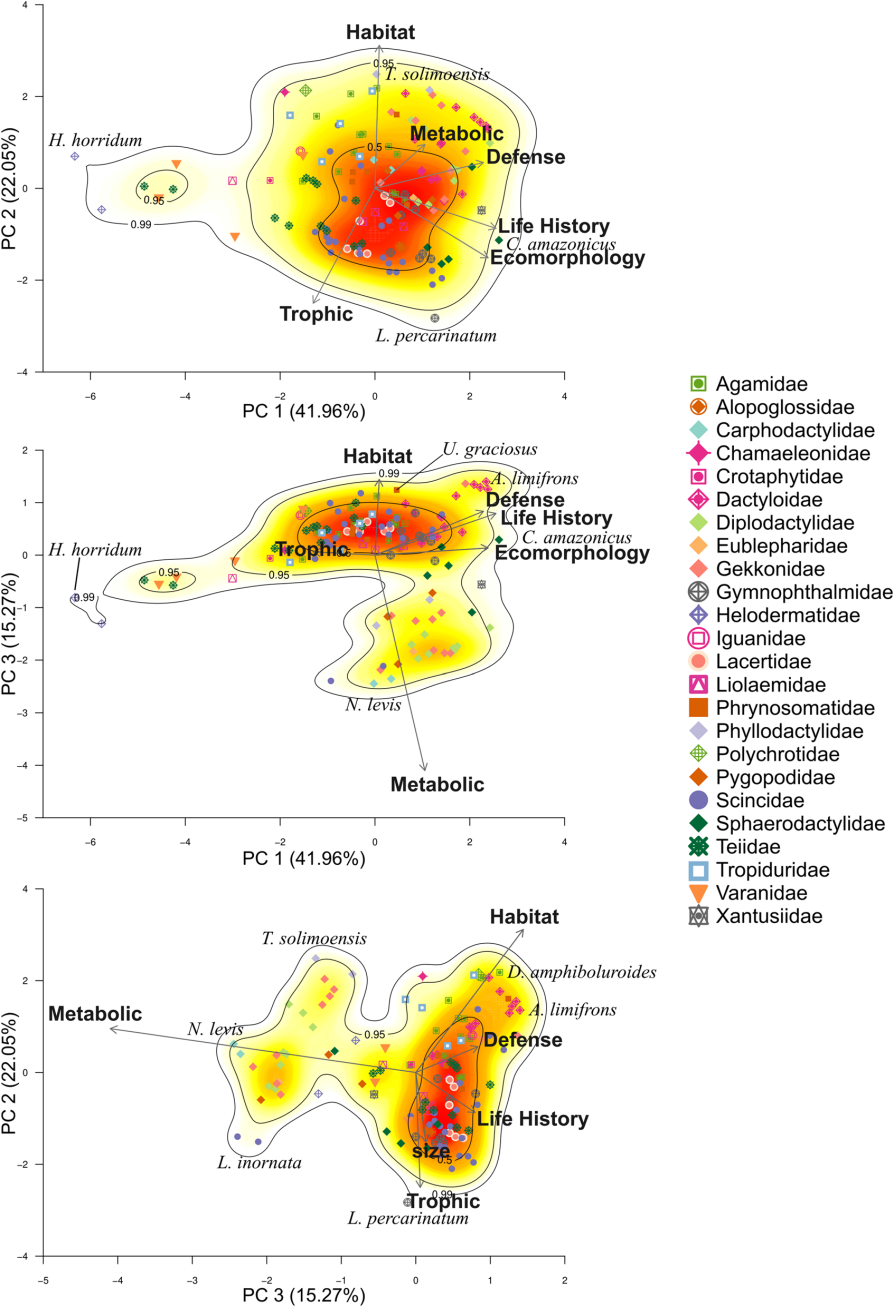
Three-dimensional view of PCA ordination based on analysis of species scores on each of six gradients extracted from PCoA performed on functional trait matrices associated with five niche dimensions and ecomorphology. Kernel Density Estimation shows areas including 50%, 95% and 99% of species

Two-dimensional Kernel Density Estimation on the first two PCA axes show one large group with density probability of 50%, with high species similarity on the trophic and metabolic dimensions (Figs. 1 and 2). Large and aggressive species (e.g., *Varanus*, *Heloderma*, and the tegus) and species with large clutches (e.g., *Salvator merianae*, *Chamaeleo chameleon*), and small species with passive defense strategies and small clutch size (like some geckos, gymnophthalmids and anoles) are located at opposite ends of PC1. Species are positioned along PC2 mainly according to foraging mode and substrate used. Thus, arboreal, ambush foragers (e.g., *Thecadactylus solimoensis*, *Polychrus acutirostris* and *Chamaeleo chamaeleon*) are positioned opposite to fossorial, ground-dwelling and widely foraging species (e.g., *Loxopholis percarinatum* and *Menetia greyii*). When analyzing PC1 vs PC3, at least two groups can be recognized based on activity time. Nocturnal species (e.g., *Liopholis striata*, *Xantusia vigilis*, and nocturnal geckos) are clearly separated from diurnal species. Analyzing PC3 against PC2, shows a clear separation between diurnal and nocturnal species on PC3, with species from both groups ordered along PC2 according to habitat use.

The extended database (1023 species, merging Pianka and Vitt’s with Meiri’s datasets) had representatives for ~ 92% of functional groups, only lacking Sit & Wait, Semi-Aquatic, Nocturnal (SW-Aq-N) species. The most diverse functional group was Widely Foraging, Terrestrial, Diurnal (WF-T-D) with 24 families and 401 species, followed by Sit & Wait, Terrestrial, Diurnal (SW-T-D) with 26 families and 265 species, while Sit & Wait, Cryptic, Nocturnal (SW-C-N) and Sit & Wait, Fossorial, Nocturnal (SW-F-N) were less diverse with only three species each. Scincidae was the most species-rich family of the Widely Foraging, Terrestrial, Diurnal (WF-T-D) group (118 species, out of 1709) extant species described), and Agamidae was the family with most species from the Sit & Wait, Terrestrial, Diurnal (SW-T-D) group, with 46 out of 530 extant species. Scincidae was functionally the most diverse family, with representatives for 19 functional groups, followed by Sphaerodactylidae with species in 13 functional groups, Anguidae (12 functional groups), and Gekkonidae and Phyllodactylidae (11 functional groups). The rest of lizard families had representatives for eight or less functional groups, with Shinisauridae belonging to only one functional group. Species belonging to more than one functional group were frequent (432 out of 1023 species, ~ 42%), especially in Gekkota (~ 56% of species) (Additional file 1).

**Fig. 3:**
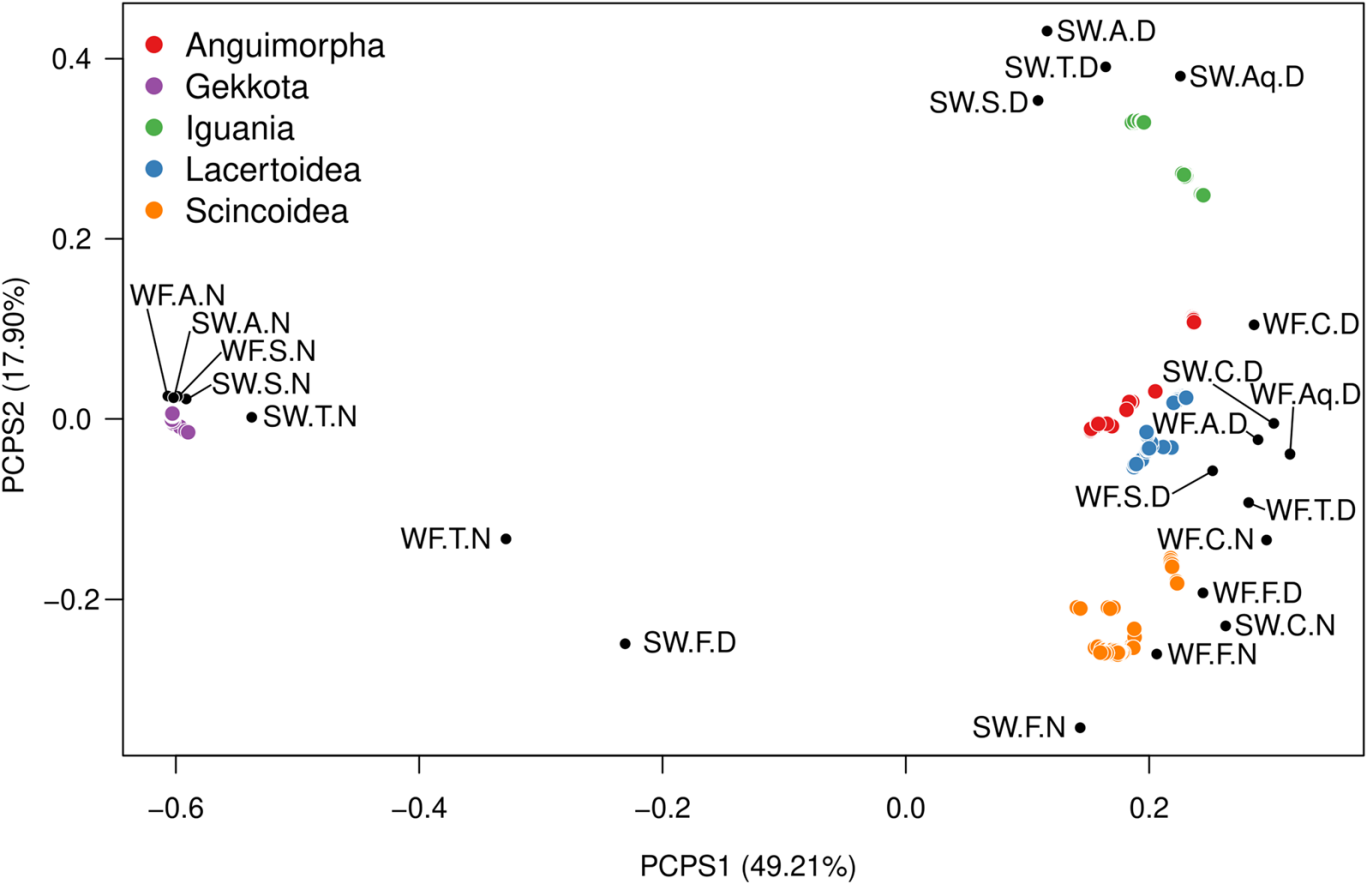
Scatter diagram of the two first axes generated from principal coordinates of phylogenetic structure (PCPS) for 1023 lizard species occurring in functional groups determined by foraging mode, substrate, and activity. Dot colors indicate membership in large lizard clades. Black dots indicate the centroid for each functional group

Permutation tests under null model testing the hypothesis of no phylogenetic structure indicate strong phylogenetic clustering for functional groups ($\overline{PSV }obs$ = 0.6545; $\overline{PSV }null$ = 0.8620, confidence interval: 0.828–0.881; p null < 0.05). The first PCA axes from Phylogenetic Structure (PCPS) captured 49.2% of variance and separated diurnal and nocturnal species (the latter were almost exclusively gekkotans), with the exception of Widely Foraging, Cryptic, Nocturnal (WF-C-N) and Widely Foraging, Fossorial, Nocturnal (WF-F-N), which clustered along with diurnal species and were composed of nocturnal skinks. Thus, PCPS1 reflected a basal split between Gekkota and the rest of squamates. PCPS2 (17.9% of variance) separated lizards mainly according to clades associated with either sit-and-wait or widely foraging hunting strategies. The highest values of PCPS2, correspond to functional groups dominated by Iguania, all of them diurnal, cursorial, and ambush foragers. Functional groups associated with medium–low scores on PCPS axis 2 formed a cluster dominated by Anguimorpha, Lacertoidea, and Scincoidea species that are cursorial and fossorial widely foragers (diurnal and nocturnal), and fossorial ambush foragers. Nocturnal geckos clustered tightly, near the center of PCPS axis 2 (Fig. 3).

## Discussion

The observed lizard niche hypervolume was significantly more constrained than those generated by all proposed null models. Given the heterogeneous nature of the species pool included, this may indicate the influence of mixed factors such as habitat filtering, competition, or convergence, among others [21]. According to the null model most similar to the observed data (null model 4), species tend to be normally distributed along functional trait gradients, and therefore certain functional groups would be expected to have more species than others, and some trait combinations may be absent. For example, Sit & Wait, Terrestrial, Diurnal (SW-T-D) and Widely Foraging, Terrestrial, Diurnal (WF-T-D) included more species and families than other functional groups, functional groups that included fossorial species had relatively few species, and sit & wait-aquatic-nocturnal species were absent. Missing combinations of traits are likely not adaptive, and would be responsible for the reduced functional volume.

Activity time and foraging mode had a strong influence on the composition of the largest functional groups. Within these large groups, lizard species segregated by habitat, creating a complex arrangement of 23 subgroups out of 24 possible combinations. The observed reduction of a high dimensional space into a smaller set of niche dimensions occurred because certain traits were strongly intercorrelated. We found strong correspondence between ecomorphology, defense, and life history gradients (niche dimensions, each defined by sets of associated functional traits) with PCA axis 1 (PC1) (Additional file 2). Large and aggressive species (e.g., *Salvator merianae*, *Varanus gouldii*, and *Heloderma suspectum*) and species with large clutch sizes (e.g., *Salvator merianae* and *Chamaeleo chameleon*), and small and cryptic species with small clutch sizes (e.g., gymnophthalmids, alopoglossids, and some skinks) are positioned at opposite extremes of PC1 (Fig. 1). PC2 was strongly influenced by species values on the trophic dimension (i.e., diet), and species with contrasting metabolic strategies (ambush foragers vs. active foragers) were positioned at opposite extremes of this gradient. PC3 was strongly influenced by the activity/metabolic dimension, with nocturnal and diurnal species positioned at opposite extremes of this gradient. Ecomorphological variables seem to have a stronger influence on species distribution along PC1 than habitat (substrate) use. However, habitat use did influence distribution of species along PC2, with arboreal species having higher scores and fossorial species having lower scores along this axis.

Despite widespread evidence of niche convergence, phylogeny clearly influenced functional group composition. Species in most functional groups were significantly more related to each other than expected by chance. Twenty five out of the 35 families included in our extended, global dataset were represented by five or fewer functional groups, suggesting that these families had undergone limited adaptive radiation, or else had evolved into a greater number of niches but then representatives of some niches subsequently went extinct, or both. For example, dactyloids and agamids are mostly arboreal, ambush foragers and diurnal, geckos are mostly nocturnal, and lacertids are mostly terrestrial, active roamers and diurnal. However, this does not mean that all species within a family exploit resources or use habitat in exactly the same manner. Some family members use resources in different ways, with some species convergent with species from other families (e.g., *Lygodactylus klugei*—a diurnal, arboreal gecko—and dactyloids). This is especially noticeable in families with large numbers of species.

The number of species and the number of functional groups within a family were strongly and positively correlated (r = 0.77, P < 0.0001). Thus, families with less than 10 species had fewer than five functional groups, whereas more species-rich families, such as Scincidae (191 spp. and 13 functional groups), had more (Additional file 3). Although this evolutionary trend seems straightforward, differences in dispersal capabilities may be a more plausible explanation for disparity in the number of functional groups among families. Widespread families, such as Scincidae, occupy a large proportion of the lizard functional hypervolume. The Scincidae originated in Southeast Asia and apparently were adapted to a wide range of environmental conditions, allowing them to disperse globally [22]. The Agamidae also originated in Southeast Asia [23] and later dispersed into Africa and Australia. Yet the Agamidae had only seven functional groups that were restricted to a small niche hypervolume that included both diurnal and cursorial activity. In general, the largest functional groups had many representatives from the most species-rich and widespread lizard families (Additional file 1), with high levels of convergence among them.

Niche generalists, such as *T. hispidus*, have traits shared with species from several functional groups, and generalists usually were positioned in the interstitial spaces between kernel clusters. Because they often occupy diverse habitats, generalist lizards sometimes function as trophic links in spatially structured food webs [24]. This is because generalist species encounter diverse prey and predators as they move throughout various regions of heterogeneous habitats. Similarly, cathemeral species that are active during both day and night (e.g., *Delma* and *Lialis—*Pygopodidae, and *Xantusia—*Xantusiidae) link temporal food web compartments [25]. Notably, *Tarentola mauritanica*, an invasive phyllodactylid gecko from northern Africa and southern Europe [26], was the species belonging to the greatest number of functional groups. This gecko is both saxicolous and arboreal, has mixed foraging strategies, and an extended activity period that includes both day and night.

Positions of functional groups within the Principal Components of Phylogenetic Structure (PCPS) ordination space reveal phylogenetic influence on the composition of functional groups. Functional groups positioned close together in the PCPS space tend to be more closely related, with degree of relatedness represented in the various PCPS axes. Functional group composition was mainly associated with the basal split between Gekkota and the rest of Squamata, as seen in PCPS axis 1. Nocturnality evolved early in squamates, and it is the dominant feature in gekkotans [27–29]. PCPS clearly shows this split both in squamate phylogeny and in the evolution of lizard activity, with all functional groups dominated by nocturnal geckos in one extreme of PCP axis 1, and two nocturnal functional groups only composed by skinks plus all diurnal functional groups in the opposite extreme of this axis. PCPS axis 2 separates Iguania from Anguimorpha, Lacertoidea and Scincoidea, mainly representing the sit-and-wait vs. widely foraging dichotomy. Lower values on PCPS axis 2 represent an increase in the proportion of widely foraging species in functional groups. Anguimorpha, Lacertoidea and Scincoidea—although also having some ambush foraging species (29%, 11%, and 21% respectively)—formed a large cluster dominated by widely foraging species. Ambush foraging was likely the condition of ancestral Lepidosauria, a state retained in gekkotans and secondarily acquired in Iguania and some members of other major clades (e.g., Xenosauridae—Anguimorpha, Cordylids—Scincoidea, and some Lacertids—Lacertoidea) [30, 31]. The position of Gekkota and nocturnal functional groups in PCPS axis 2 clearly represents the mixture of foraging strategies used by different species of geckos.

A reliable association of any clade to a functional group will depend on how well the full spectrum of functional traits is represented in the dataset for that clade, and the accuracy of information. Given that we cannot know if accurate values for every trait were included in our extended dataset (information about autecology of most lizards is currently incomplete), results should be analyzed with a degree of caution. The proportion of described species included in this study for each family can be found in Additional file 4.

The observed degree of niche convergence or conservatism provides insight into patterns and processes of adaptation, with some niches being more evolutionarily constrained than others. Our analysis revealed clear examples of both niche convergence and conservatism. Some lizard families revealed relatively low variation for certain traits, such as clutch size in gymnophthalmids and terrestrial habitat use in teiids. These cases are assumed to represent ancestral state retention, the essential feature of phylogenetic niche conservatism.

The Teiidae originated in the Northern Hemisphere and were quite diverse during the Cretaceous. This lineage colonized South America during the Cretaceous-Tertiary transition and later went extinct in the North. Teiids recolonized North America during the Late Miocene, and intervals of connection and isolation between North and South America led to the present-day distributions of teiid genera [32]. Throughout this long history of speciation and dispersal across two continents, teiids appear to have retained their ancestral niche, occupying similar habitats across a broad geographic distribution. The great majority of teiids are diurnal, terrestrial, and widely foraging, but a few species evolved semi-arboreal (*Kentropyx*) or semi-aquatic (*Dracaena* and *Crocodilurus*) habits in tropical environments.

Identification of convergent species based on a reduced number of functional variables is useful for addressing some urgent questions in conservation biology. For example, Jarnevich et al*.* [33] assessed the invasive potential of Neotropical tegu lizards (*Salvator* and *Tupinambis*) for different areas of North America using species distribution models. The modeled distribution revealed areas with potentially suitable habitat, and under an assumption of niche conservatism, these lizards would be capable of establishing populations if introduced in North America. No large predatory lizards have existed in North America since the extinction of polyglyphanodonts (Cretaceous–Paleogene Mass Extinction—K–Pg event, ~ 65 mya) and varanoid-like platynotans (Eocene ~ 35 mya) [34, 35]. In fact, only small and medium-sized lizards were found in the Paleogene after the K–Pg event, a pattern maintained until today [35]. Given the possibility of an empty niche created by the extinction of polyglyphanodonts and varanoid-like platynotans, recent introductions of large lizards like tegus (*Salvator merianae*) and Nile Monitors (*Varanus niloticus*) [36] in the southeastern United States are likely to be successful with serious implications for conservation of native biota. Following the K–Pg event, North American lizards evolved without lizards as large as tegus and Nile Monitors [36]. If tegus and monitor lizards are indeed convergent with polyglyphanodonts and playnotans, then knowledge of their ecology in invaded regions might allow us to infer ecological interactions that occurred within ancient lizard communities. Much can be inferred about the ecology of extinct species that are convergent with extant species on one or more niche dimensions. For example, based on its distinct functional traits, *Leedsichthys problematicus*, a giant ray-finned fish known from fossils dating to the Upper Jurassic, is convergent with some of the largest modern-day elasmobranchs that are filter feeders, including the Whale shark, Basking shark, and Megamouth shark [37]. This allows researchers to hypothesize not just about the feeding habits of extinct species, but also about their physiological, behavioral and evolutionary ecology [38, 39].

Using ecological data for lizards compiled by Meiri [40], Vidan et al*.* [41] performed archetypal analysis to identify seven principal ecological strategies: scansorial, terrestrial, nocturnal, herbivorous, fossorial, large, and semi-aquatic. Based on analysis of both quantitative and qualitative variables, our classification of functional groups included most of the ecological strategies recovered by Vidan et al*.* [41], although with some differences in group composition. Archetypal analysis creates clusters and calculates the probability of group membership for each species. Some of the nocturnal species included in Vidan et al*.*’s analysis ended up being grouped outside the nocturnal strategy; in those cases, other traits had more weight than activity time in the archetypal analysis. In the present work, all nocturnal species were accurately assigned to one of the “nocturnal” groups defined by the combination of activity with foraging mode and habitat to yield a more reliable classification.

Methods adopted here are suitable for organizing species according to an ecological classification scheme analogous to periodic tables of niches [19, 20]. As we demonstrated here, ordination and classification of ecological niches summarizes knowledge of species ecology, yields insights into evolutionary processes, and promotes generation of new hypotheses. Widespread convergences revealed here suggest that many lizard species throughout the world have independently evolved to play the same role in Elton’s ecological play. In some sense, this concept of niche evolution contrasts with species-centric niche concepts. Similar *n*-dimensional niches can be occupied by species from different lineages in different parts of the world, such as a tropidurid lizard in South America and a cordylid in Africa [42]. Furthermore, if the temporal dimension is considered, we can hypothesize that the same niche might have been occupied by some extinct saurian species—probably one of the highly diversified Rhynchocephalia during the mid-Triassic [43]. Extensive field work will be required to fill existing data gaps, improve data resolution, and expand phylogenetic coverage for a more thorough analysis of global niche diversification and convergence in lizards. Larger and higher resolution datasets will undoubtedly achieve a broader and deeper understanding of how niches have evolved within various lineages and regions of the Earth.

## Conclusions

The present study expands methods and ideas presented in previous analyses of lizard niche diversity [19, 41]. While previous research has examined patterns in the distribution of lizard species along niche dimensions [19] or how groups of ecologically similar species are distributed globally [41], the present study explored the manner and extent to which lizard functional trait combinations are constrained by ecological and phylogenetic factors, and how these factors determine the composition of functional groups. Comparison of empirical patterns with those generated by null models suggests that ecological filters promote limited sets of trait combinations, especially where similar conditions occur, reflecting both niche convergence and conservatism. We found 24 unique combinations of foraging modes, activity periods, and habitats, and one combination that was absent from our global lizard dataset (sit & wait/nocturnal/aquatic. Divergence in activity patterns tended to be associated with basal phylogenetic relationships, with most nocturnal groups dominated by gekkotans. Foraging mode and habitat tended to diverge at shallower nodes, with a large split between Iguania and all other lizard clades. The identification of species with similar functional traits or trait combinations (reflecting either conservatism or convergence) has multiple potential applications, because similarity facilitates inferences about trait values and/or ecological roles of poorly documented species. Our discovery of widespread niche convergence indicates that lizard species throughout the world have independently evolved to play the same role in habitats with similar environmental conditions. In some sense, this concept of niche evolution contrasts with species-centric niche concepts.

## Methods

### Data

This study analyzes a dataset of ecological variables for 1023 lizard species, representing 35 of the 44 recognized extant families from every biogeographical realm where lizards are present (Neartic, Paleartic, Neotropic, Afrotropic, Indomalasyan, Australasia, and Oceania, see Additional file 2). The dataset was constructed from high-resolution, ecological data for lizard species from four continents [19, heretofore referred to as Pianka and Vitt’s dataset] and a functional traits database with lower resolution that included thousands of lizard species globally [40, refered to as Meiri’s dataset]. Pianka and Vitt’s dataset includes 134 lizard species and 41 ecological variables. Meiri’s dataset includes 6657 lizard species, and 11 ecological variables. Among the 6524 species in Meiri’s dataset that were not included in Pianka and Vitt’s dataset, only 912 species had data for habitat, foraging mode, and activity period (important variables defining functional groups—see below). One species from Pianka and Vitt’s dataset—*Ctenotus inornatus*—was not in Meiri’s dataset. Combining species in Pianka and Vitt’s dataset with those having suitable and complete data from Meiri’s dataset provided at total of 1046 species. Among these, 23 species were not included in the latest phylogeny available [44] and therefore were excluded.

Categorical variables describing habitat use (five variables: Semi-aquatic, Fossorial, Terrestrial, Saxicolous, and Arboreal) from Pianka et al.’s [19] original dataset were re-coded into six binary variables to match the categories used in Meiri’s dataset (Aquatic, Fossorial, Cryptic, Terrestrial, Saxicolous, and Arboreal). Mixed or intermediate states, such as mixed foraging strategy, cathemeral activity [i.e. both diurnal and nocturnal, 45], or semi-arboreal habits, were scored for more than one variable. For example, cathemeral species were scored for both Diurnal and Nocturnal activity. Ecological variables used in [19] were grouped according to five fundamental niche dimensions as follows: habitat (Fossorial, Cryptic, Terrestrial, Saxicolous, Arboreal, Semi-Aquatic), life history (Female Weight, Female SVL, SVL at Maturity, Juvenile SVL, Clutch Size, Clutch Frequency, Reproductive Clutch Mass—RCM, Oviparous, Viviparous), trophic (dietary percentage of—Larvae, Vertebrates, Plant parts, Ants, Isopterans, Large insects, and Arachnids), metabolic (Body Temperature, Slope of Body Temperature against Air Temperature, Diurnal, and Nocturnal), and defense (Armor, Crypsis, Color Change, Tail Color, Mimicry, Thanatosis, Saltation, Autotomy, Spines, Goop, Bite, Flee, Threat, and Venom). We included ecomorphology as an additional functional trait category that reflects performance associated with more than one fundamental niche dimensions (Snout-Vent Length—SVL, Tail Length, Body Weight, Head Length, Head Width, Head Height, Forelimb Length, Hindlimb Length).

### Statistical analyses

We used Pianka and Vitt’s detailed dataset for 134 lizard species (that includes 24 of the 44 extant families from the Americas, Africa, and Australia) organized according to six dimensions to evaluate constraints to functional diversity, and to identify the principal variables defining lizard functional groups. We obtained the distribution of each of the 134 lizard species along the principal axis of ecomorphology, habitat, trophic, life history, metabolic, and defense dimensions by extracting scores on the first axis from Principal Coordinates Analyses (PCoA) performed separately on each dimensional trait dataset, using unweighted Gower distances [See details in 19]. These components are combinations of traits that modeled the greatest amount of variation for each dimension. Species scores on these six PCoA axes were then used as variables that reflected interspecific variation associated with habitat substrate, diet, reproduction, activity, defense, and morphology to facilitate interpretation of results in subsequent analyses. Following procedures described in Diaz et al. [15], we then calculated a six-dimensional hypervolume, using the package *geometry* [46] for R [47]. We used an adaptation of Cornwell et al*.*’s [48] approach to test whether the observed volume was more functionally constrained than expected under four null models of species distribution in a functional hyperspace: Null model 1—species are distributed uniformly in the functional space and independent from each other, and functional variables are not correlated; Null model 2—species are normally distributed in functional space and independent from each other, and functional variables are not correlated; Null model 3—species are distributed as observed, and functional variables are not correlated; and Null model 4—species are normally distributed in functional space, and functional variables are correlated as observed. We followed methods used by Diaz et al. [15] to build our convex hull polygons and to compare the observed volume with the four null models. Convex hulls provide a basic representation of the functional hyperspace, because they are constructed using extreme observations from each dimension [49]. Functional hypervolumes also can be estimated based on probabilistic approaches that might provide a higher resolution portrayal of species distributions as well as holes within trait hyperspace [50]. However, for accurate estimations for hypervolume size and shape, this approach requires progressively larger sample sizes as trait dimensionality increases [51]. Given the high dimensionality of our dataset (six dimensions), and the limited number of species with detailed ecological information (134 species in the Pianka and Vitt’s dataset), an accurate estimation of hypervolumes using a probabilistic approach was not possible [49]; therefore, we used convex hulls, acknowledging its limitations.

Functional variables extracted from the six PCoAs (substrate, diet, reproduction, activity, defense, and morphology) were then used as independent variables in a Principal Components Analysis (PCA) to obtain an ordination of the species in lizard niche space. We used the *factoextra* [52] and *FactoMineR* [53] packages for R [47] to run the PCA and to calculate the contribution of each functional variable to the ordination. Kernel Density Estimation [KDE, 54] was used to identify regions of highest occurrence probability for species in the 3D niche space (defined by the first three PC axes), using the package *ks* [55] for R [47], and the functional variables best related to the observed ordination. Diet, activity, and habitat were the functional variables that best grouped species within the functional space represented by the PCA with KDE plot (see “Results” section).

We then used the extended dataset (1023 species) to classify lizard species into functional groups based on combinations of activity time (Diurnal, Nocturnal), foraging strategy (Sit-and-wait vs. Widely-foraging), and habitat use (Fossorial, Cryptic, Terrestrial, Saxicolous, Arboreal, Semi-Aquatic). From the 24 possible functional groups, 23 were represented among species in the extended database (Additional file 1): Sit & Wait-Semi Aquatic-Diurnal (SW-AQ-D), Widely Foraging-Semi Aquatic-Diurnal (WF-AQ-D), Widely Foraging-Semi Aquatic-Nocturnal (WF-AQ-N), Sit & Wait-Fossorial-Diurnal (SW-F-D), Sit & Wait-Fossorial-Nocturnal (SW-F-D), Widely Foraging-Fossorial-Diurnal (WF-F-D), Widely Foraging-Fossorial-Nocturnal (WF-F-N), Sit & Wait-Cryptic-Diurnal (SW-C-D), Sit & Wait-Cryptic-Nocturnal (SW-C-N), Widely Foraging-Cryptic-Diurnal (WF-C-D), Widely Foraging-Cryptic-Nocturnal (WF-C-N), Sit & Wait-Terrestrial-Diurnal (SW-T-D), Sit & Wait-Terrestrial-Nocturnal (SW-T-N), Widely Foraging-Terrestrial-Diurnal (WF-T-D), Widely Foraging-Terrestrial-Nocturnal (WF-T-N), Sit & Wait-Saxicolous-Diurnal (SW-S-D), Sit & Wait-Saxicolous-Nocturnal (SW-S–N), Widely foraging-Saxicolous-Diurnal (WF-S-D), Widely Foraging-Saxicolous-Nocturnal (WF-S–N), Sit & Wait-Arboreal-Diurnal (SW-A-D), Sit & Wait-Arboreal-Nocturnal (SW-A-N), Widely Foraging-Arboreal-Diurnal (WF-A-D), Widely Foraging-Arboreal-Nocturnal (WF-A-N). Species with mixed strategies (Cathemeral, Mixed foraging behavior, Habitat generalists) were assigned to more than one functional group. For example, *Chalcides ocellatus* was included in the functional groups WF-C-D, WF-C-N, SW-C-D, and SW-C-N.

To assess the influence of phylogeny on the structure of functional groups, we calculated the mean phylogenetic distance of species within functional groups [Phylogenetic Species Variability—PSV, 56]. PSV ranges from 0 to 1, with values equal to 1 meaning species are maximally unrelated (overdispersed pattern), and values equal to 0 indicating maximum relatedness (a clustered pattern). PSV was calculated for each functional group containing at least two species [56]. We used permutation tests to determine whether the observed average PSV value across all functional groups ($\overline{PSV }obs$) differed from that expected under the null hypothesis. The null hypothesis assumes that functional groups consist of random draws of species from the pool of 1,023 lizard species. Therefore, it assumes no phylogenetic structure in functional group composition. For null model, we randomly extracted 1,000 samples of species from the entire species pool, calculated PSV for each run, and then created a distribution of PSV values. Randomized values between quantiles $\alpha /2$ (0.025) and $1-\alpha /2$ (0.975) were thus calculated and used as lower and upper limits for significance. Rejecting the null hypothesis means that functional groups are not a random sample of species from the species pool. Thus, $\overline{PSV }obs$ < 0.025 quantile indicates that species belonging to a functional group are more closely related with each other than expected by chance—a clustered pattern, whereas $\overline{PSV }obs$ > 0.975 quantile indicates that species within functional groups are unrelated—an overdispersed pattern [56]. Phylostructure was tested using the function phylostruct from the *Picante﻿* package [57] for R [47].

To assess how phylogeny explains differences in species composition between functional groups, we estimated phylobetadiversity and then principal coordinates of phylogenetic structure (PCPS) [58, 59]. Phylobetadiversity was implemented using Phylogenetic Fuzzy Weighting (PFW), a method intended to analyze phylobetadiversity patterns across biological communities or metapopulations [58–60]. PFW uses pairwise phylogenetic similarities between species to weight their occurrence in a functional group, resulting in a species-by-functional group matrix (matrix P). The advantage of PFW over other methods (COMDIST, COMDISTNT, Rhao’s H, UniFrac, etc.) is that PFW is likely to capture phylobetadiversity patterns associated with both basal and terminal phylogenetic nodes, whereas other methods will likely find patterns associated with either basal or terminal nodes [59]. Moreover, PFW allowed us to decompose phylogenetic gradients across the array of functional groups into orthogonal eigenvectors and, more importantly, to evaluate which clades are related to each phylogenetic eigenvector [59–61].

To evaluate relationships between clades and phylogenetic eigenvectors, we performed a PCoA based on the square-rooted Bray–Curtis dissimilarities between pairs of functional groups previously computed on matrix P. The resulting eigenvectors represented principal coordinates of phylogenetic structure (PCPS). The PCPS with the largest eigenvalue describes broader phylogenetic gradients related to the split of the deepest tree nodes across the dataset, such as the one connecting Gekkota and the rest of Squamata. As the eigenvalues of the other PCPS axes decrease, finer phylogenetic gradients related to splits of shallower nodes (e.g., families, genera) are described [60]. PCPS axes with percentage of explained variance > 10% were retained for interpretation of grouping patterns among functional groups [62]. PCPS were calculated using the package *PCPS* 1.07 [63] for R [47].

## Supplementary Information

**Additional file 1.** A: Map showing localities for species included in this work. B: Phylogenetic hypothesis used, following Tonini et al. [44].

**Additional file 2.** Correlation patterns among Principal Component axes, reflecting correlation between functional variables (i.e. ecomorphology, habitat, trophic, life history and defense).

**Additional file 3.** List of species included in this study, detailing functional group membership for each species.

**Additional file 4.** Proportion of described species included in this study for each family and Clade. Number of described species according to The Reptile Database (Uetz et al. 2020) [26].

## Data Availability

All data generated or analyzed during this study are included in these published articles and its supplementary information files: Ref [19, 40].
