# Supplementary figures and images for: How do lizard niches conserve, diverge or converge? Further exploration of saurian evolutionary ecology
Source: BMC Ecol Evol. 2021 Jul 30;21:149. doi: 10.1186/s12862-021-01877-8 (PMC8323276; doi:10.1186/s12862-021-01877-8)

A

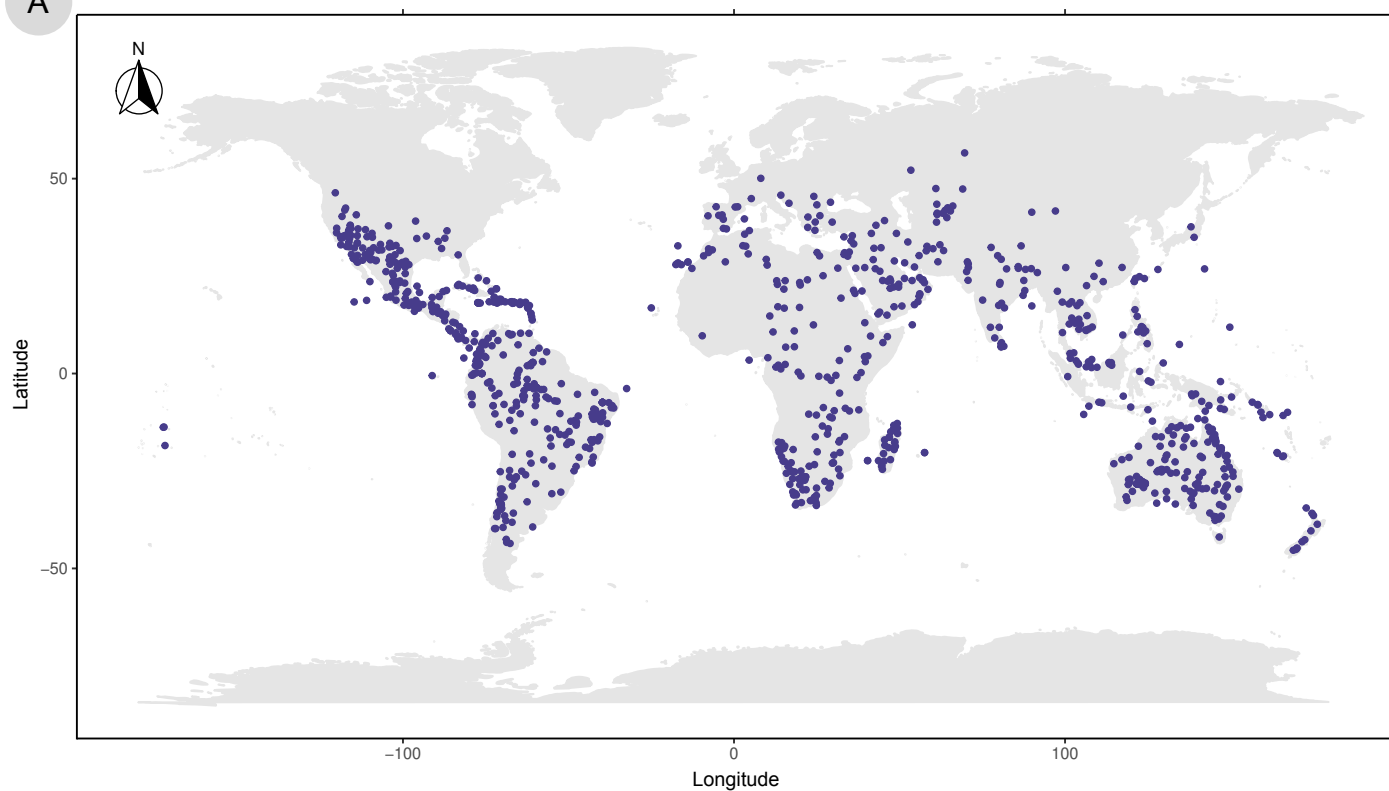

B

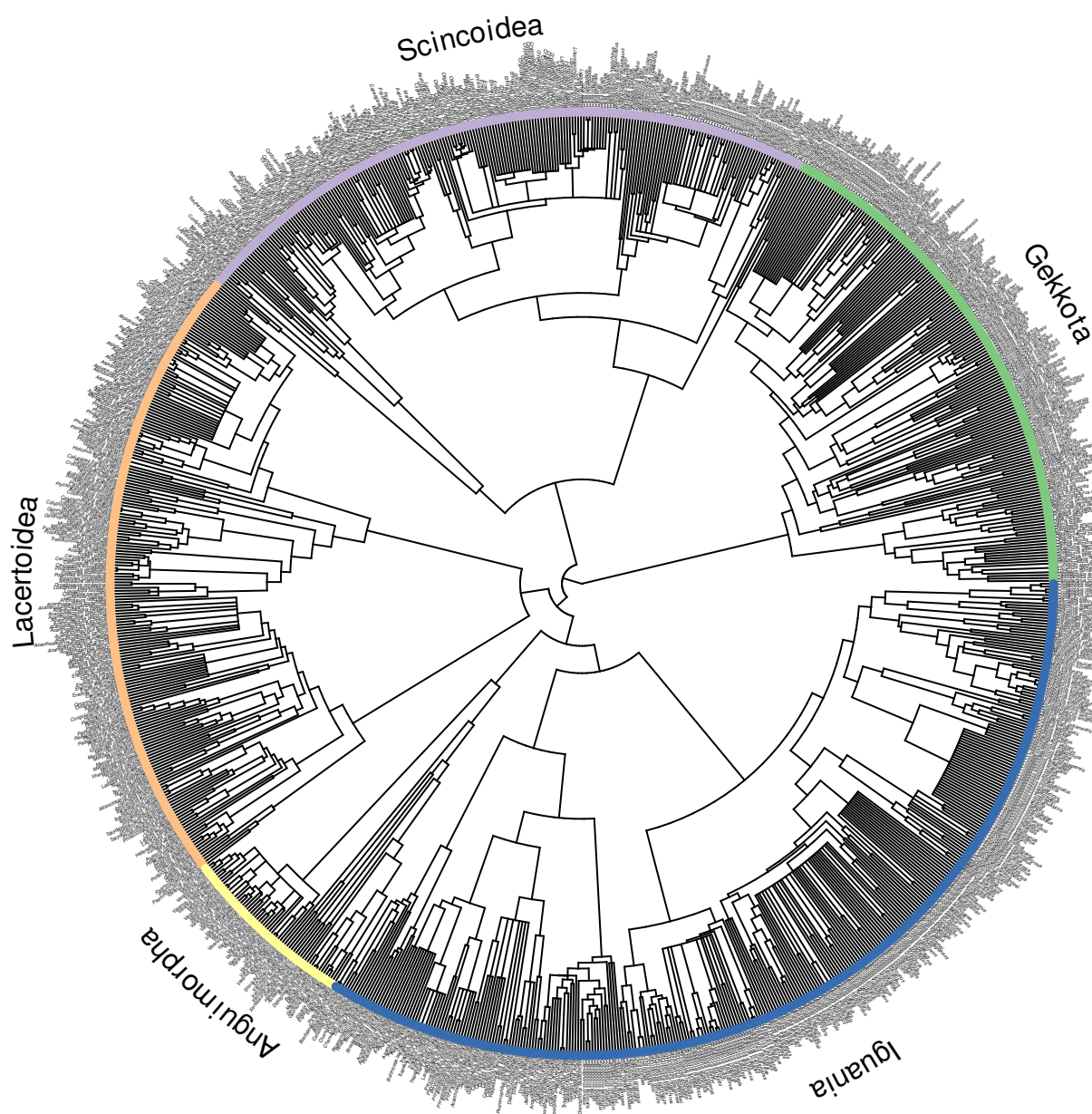

Supplement: Supplementary file 1 — Additional file 1. A: Map showing localities for species included in this work. B: Phylogenetic hypothesis used, following Tonini et al. [44]. [file 12862_2021_1877_MOESM1_ESM.pdf]

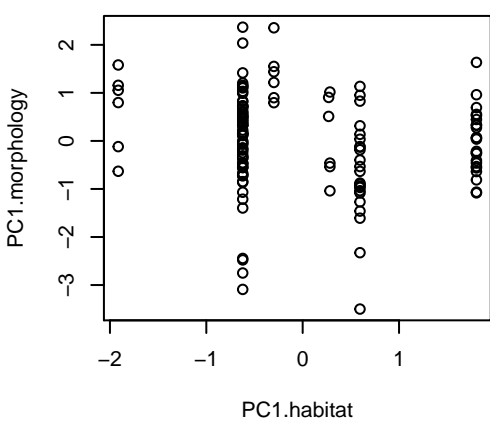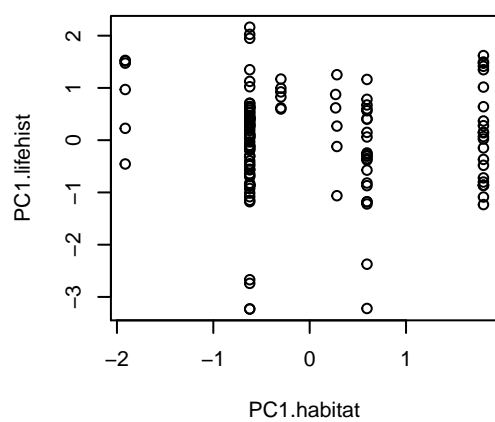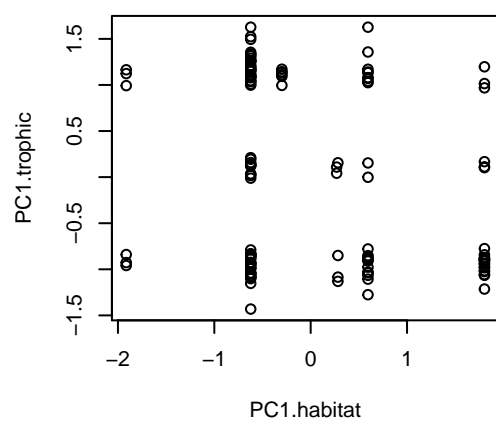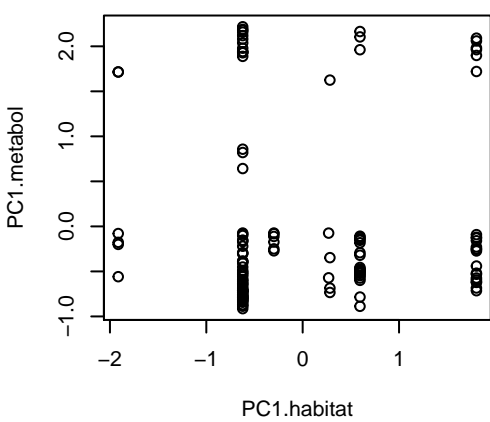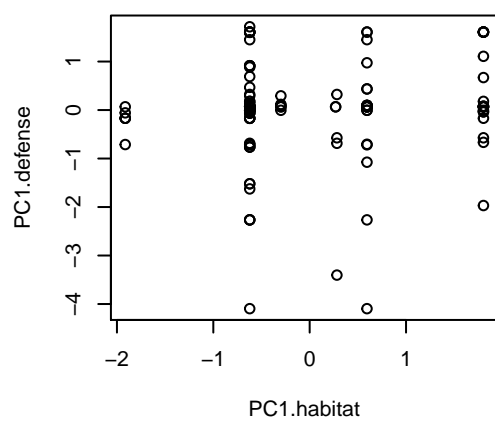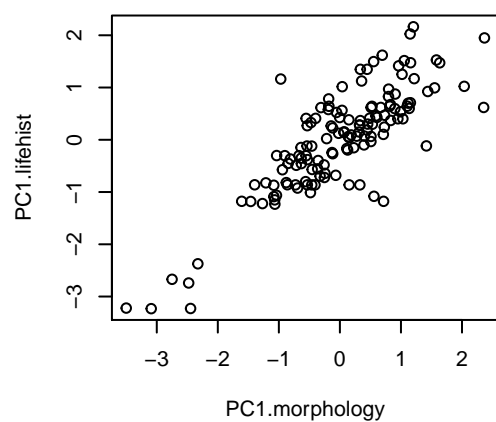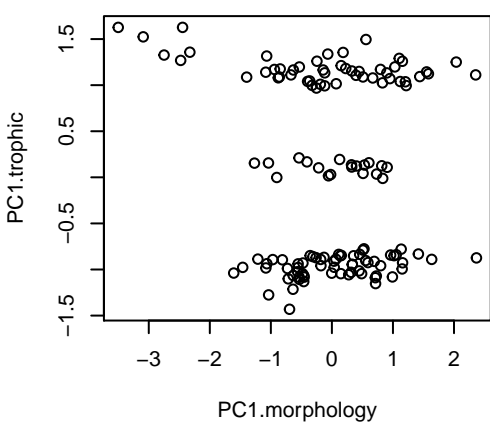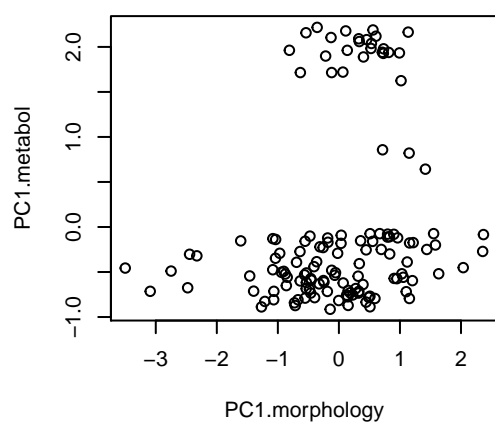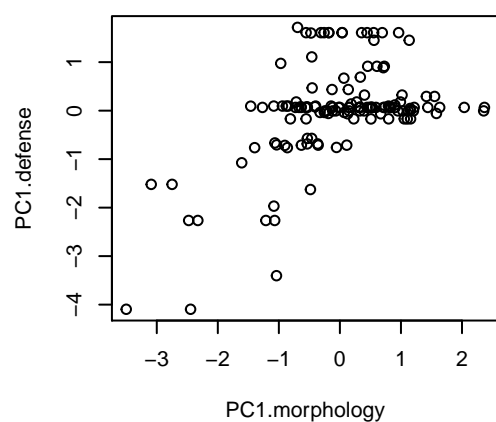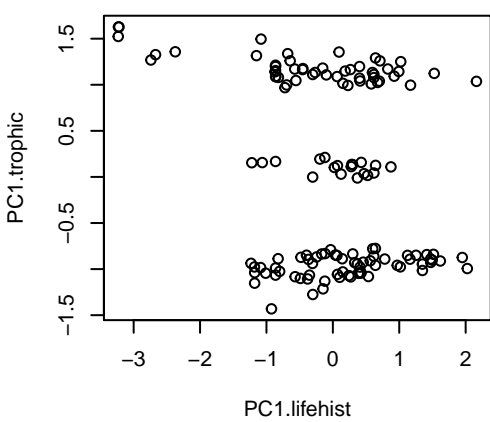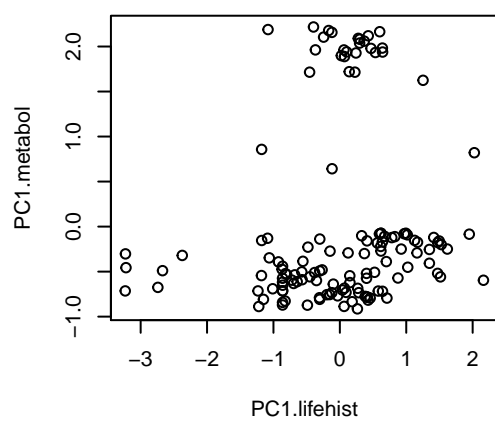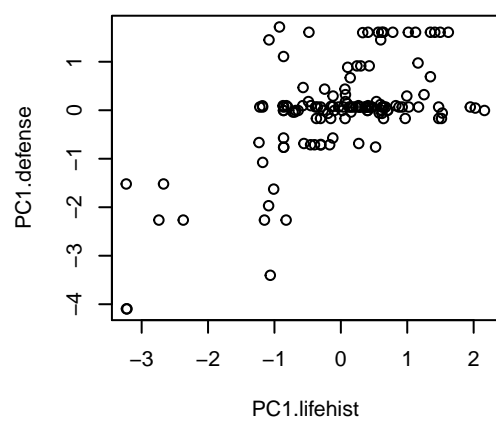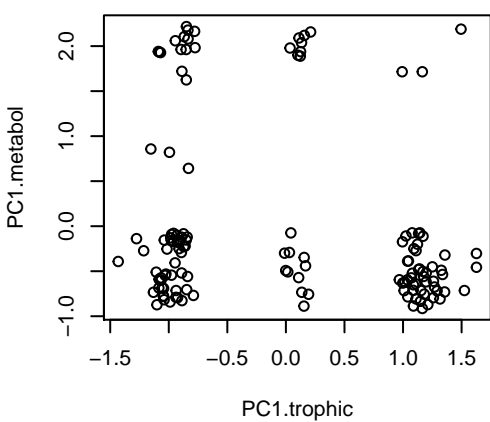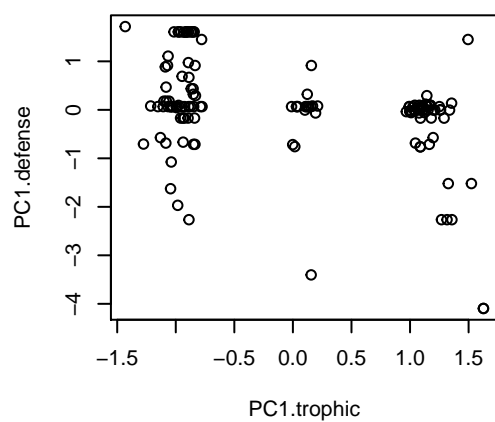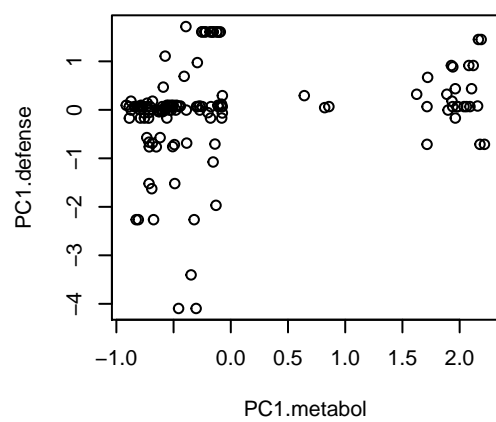

Supplement: Supplementary file 2 — Additional file 2. Correlation patterns among Principal Component axes, reflecting correlation between functional variables (i.e. ecomorphology, habitat, trophic, life history and defense). [file 12862_2021_1877_MOESM2_ESM.pdf]
